# Electroacupuncture promotes neurogenesis in the dentate gyrus and improves pattern separation in an early Alzheimer's disease mouse model

**DOI:** 10.1186/s40659-023-00472-z

**Published:** 2023-12-02

**Authors:** Yanyi Ding, Long Li, Sinuo Wang, Yajun Cao, Minguang Yang, Yaling Dai, Huawei Lin, Jianhong Li, Yulu Liu, Zhifu Wang, Weilin Liu, Jing Tao

**Affiliations:** 1 The Institute of Rehabilitation Industry, Fujian University of Traditional Chinese Medicine, Fuzhou, Fujian 350122 China; 2 College of Rehabilitation Medicine, Fujian University of Traditional Chinese Medicine, Fuzhou, Fujian 350122 China; 3 National-Local Joint Engineering Research Center of Rehabilitation Medicine Technology, Fuzhou, Fujian 350122 China; 4 Fujian Key Laboratory of Cognitive Rehabilitation, Affiliated Rehabilitation Hospital of Fujian University of Traditional Chinese Medicine, Fuzhou, 350001 China; 5 Fujian Key Laboratory of Rehabilitation Technology, Affiliated Rehabilitation Hospital of Fujian University of Traditional Chinese Medicine, Fuzhou, 350001 China; 6 Provincial and Ministerial Co-founded Collaborative Innovation Center of Rehabilitation Technology, Fujian University of Traditional Chinese Medicine, Fuzhou, 350122 China

**Keywords:** Alzheimer’s disease, Electroacupuncture, Memory discrimination, Pattern separation, Immature granule cells

## Abstract

**Background:**

Impaired pattern separation occurs in the early stage of Alzheimer’s disease (AD), and hippocampal dentate gyrus (DG) neurogenesis participates in pattern separation. Here, we investigated whether spatial memory discrimination impairment can be improved by promoting the hippocampal DG granule cell neogenesis-mediated pattern separation in the early stage of AD by electroacupuncture (EA).

**Methods:**

Five familial AD mutations (5 × FAD) mice received EA treatment at Baihui and Shenting points for 4 weeks. During EA, mice were intraperitoneally injected with BrdU (50 mg/kg) twice a day. rAAV containing Wnt5a shRNA was injected into the bilateral DG region, and the viral efficiency was evaluated by detecting Wnt5a mRNA levels. Cognitive behavior tests were conducted to assess the impact of EA treatment on cognitive function. The hippocampal DG area Aβ deposition level was detected by immunohistochemistry after the intervention; The number of BrdU^+^/CaR^+^ cells and the gene expression level of calretinin (CaR) and prospero homeobox 1(Prox1) in the DG area of the hippocampus was detected to assess neurogenesis by immunofluorescence and western blotting after the intervention; The gene expression levels of FZD2, Wnt5a, DVL2, p-DVL2, CaMKII, and p-CaMKII in the Wnt signaling pathway were detected by Western blotting after the intervention.

**Results:**

Cognitive behavioral tests showed that 5 × FAD mice had impaired pattern separation (*P* < 0.001), which could be improved by EA (*P* < 0.01). Immunofluorescence and Western blot showed that the expression of Wnt5a in the hippocampus was decreased (*P* < 0.001), and the neurogenesis in the DG was impaired (*P* < 0.001) in 5 × FAD mice. EA could increase the expression level of Wnt5a (*P* < 0.05) and promote the neurogenesis of immature granule cells (*P* < 0.05) and the development of neuronal dendritic spines (*P* < 0.05). Interference of Wnt5a expression aggravated the damage of neurogenesis (*P* < 0.05), weakened the memory discrimination ability (*P* < 0.05), and inhibited the beneficial effect of EA (*P* < 0.05) in AD mice. The expression level of Wnt pathway related proteins such as FZD2, DVL2, p-DVL2, CAMKII, p-CAMKII increased after EA, but the effect of EA was inhibited after Wnt5a was knocked down. In addition, EA could reduce the deposition of Aβ plaques in the DG without any impact on Wnt5a.

**Conclusion:**

EA can promote hippocampal DG immature granule cell neogenesis-mediated pattern separation to improve spatial memory discrimination impairment by regulating Wnt5a in 5 × FAD mice.

## Introduction

People with Alzheimer's disease (AD) often have difficulty accurately identifying similar events [1], such as where they parked today and where they parked yesterday. This is because the two kinds of information have overlapping components (the same car), which interfere with each other in the process of memory. Normally, our brains encode this information separately through a process called pattern separation that refers to reducing overlap among similar inputs to avoid interference [2]. Relevant research findings indicate that the decline in pattern separation ability is associated with advancing age in physiological states, while pathological damage further exacerbates this phenomenon [3, 4]. In spatial discrimination tasks that rely on pattern separation, both mild cognitive impairment (MCI) and AD patients showed reduced pattern separation ability compared with cognitively normal adults, and patients with AD had the weakest ability [5–7].

The DG is considered as a “pattern separator” [8]. The subgranular zone is one of the brain regions that retains the capacity for neurogenesis throughout adulthood [9]. In human beings, about 700 newborn granule cells (GCs) are produced every day and added to the neural circuit to exert the plasticity of the hippocampus structure and function [10]. Neurogenesis is believed to contribute to pattern separation through two distinct pathways. Firstly, immature GCs solely encode input information. The immature GC is characterized by high excitability, synaptic plasticity, and insensitivity to GABA neurotransmitters, which allows it to respond to small changes in input information through rate remapping. The second is that mature GCs encode input information, while immature GCs reduce redundant encoding by mature GCs. When similar environmental stimuli are presented, the activation of immature GCs precedes that of mature GCs, and they subsequently inhibit the already encoded information in mature GCs by recruiting local inhibitory networks. This approach effectively minimizes representation overlap resulting from repetitive coding by mature GCs [11–13]. In any case, the involvement of newborn neurons is crucial, and it has been extensively established that promoting neurogenesis enhances pattern separation [14–16].

The level of neurogenesis in AD can be influenced by multiple factors, including the phosphorylation level of Tau, activity of α secretase, metabolites of APP, and the Wnt family, all of which are discussed in these reviews [17–19]. The Wnt signaling pathway is a well-established regulatory mechanism in AD neurogenesis, involving various ligands such as Wnt1, Wnt3, Wnt3a, Wnt7a, and Wnt8. These ligands have been proposed to enhance neurogenesis and cell differentiation through the canonical Wnt/β-cateinin signaling pathway. Additionally, non-canonical pathways including the Wnt/Ca^2+^ or Wnt/PCP pathways mediated by ligands like Wnt1, Wnt5a and Wnt11 ligands can influence neuronal morphological development. Interestingly, recent studies have revealed the involvement of Wnt5a in both canonical and non-canonical Wnt signaling pathways. Expression of Wnt5a was observed in postnatal day 10 rats, predominantly within the DG and CA1 regions [20], with peak expression occurring during adulthood [21]. Both exogenous Wnt5a ligand and Wnt7a exhibited a comparable effect in augmenting neurogenesis within the DG region of rats [22]. Inhibition of Wnt5a expression lead to a reduction in the quantity and compromised morphological development of newborn neurons [23]. Therefore, we demonstrated a significant interest in investigating the potential pivotal role of Wnt5a in pattern separation.

EA has been demonstrated to promote neurogenesis in various disease models by regulating the Notch signaling pathway and promoting endogenous neural stem cells differentiation in rats with cerebral ischemia–reperfusion [24]. EA was found to promote hippocampal neurogenesis, enhance synaptic plasticity, and alleviate spatial memory impairment by activating BDNF/Tr-kB/ERK signaling in sleep-deprived rats [25]. The present study has been demonstrated that EA exerts a neurogenic effect in the subventricular zone, thereby ameliorating motor dysfunction in rat with Parkinson's disease. In addition, EA was found to improve working memory by inhibiting synaptic degeneration and neuroinflammation in mouse models of AD [26]. However, it remains uncertain whether EA could serve as a potential strategy for promoting neurogenesis to enhance pattern separation in early AD model mice.

In this study, we first evaluated memory discrimination and neurogenesis in the DG area in 3-month-old 5 × FAD mice. Next, we examined whether Wnt5a plays a role in pattern separation by injecting an adeno-associated virus that interferes with Wnt5a expression into the hippocampal DG region. We further examined the memory discrimination ability and neurogenesis after 4 weeks of EA intervention to find out whether EA could improve pattern separation by promoting neurogenesis and its underlying mechanisms.

## Materials and methods

### Animals

Male 5 × FAD mice (stock #34848, Qianbi Biotechnology Co., Ltd., China) and female C57BL/6 wild-type mice were bred, and the offspring genotyping was performed by polymerase chain reaction analysis of tail DNA. The primers used included APP (oIMR 3610: AGGACTGACCACTCGACCAG, oIMR 3611: CGGGGGTCTAGTTCTGCAT), PS1(oIMR 1644: AATAGAGAACGGGCAGGAGCA, oIMR 1645: GCCATGAGGGCACTAATCAT). Both male and female mice were used in this study, and they were housed in same-sex of 3–5/cage under a 12 h light–dark cycle (lights on at 8:00 am) with access to food and water at a consistent ambient temperature (21 ± 1 °C). All experimental protocols and animal handling procedures were conducted according to the Guide for the Care and Use of Laboratory Animals published by the National Institutes of Health. This study was approved by the Ethical Committee on Animal Experimentation, Fujian University of Traditional Chinese Medicine (FJTCMIACUC2019031).

### Experimental design

This study was divided into two parts. 3-months-old 5 × FAD female and male mice were randomly divided into five groups (n = 10/group):the 5 × FAD group, 5 × FAD + EA group(5 × FAD + EA), 5 × FAD + Wnt5a interference virus group(5 × FAD + shWnt5a), 5 × FAD + EA + Wnt5a interference virus group(5 × FAD + EA + shWnt5a) and 5 × FAD + EA + empty virus group(5 × FAD + EA + shC) in each part,. Moreover, age and sex-matched littermate WT mice were classified as the WT group. Mice were evaluated for cognitive behavioral tests, including open field test (OFT), Object Location Task (OLT), Touch Screen Unique Nonmatching to Location (TUNL) task.

### Virus injection

All viruses were obtained from BrainVTA Co., Ltd. (Wuhan, China), and refer to the literature to determine the program of interfering with virus sequence and injection [23]. The rAAV-U6-DIO-shRNA (Wnt5a)-CMV-EGFP-SV40 polyA virus were injected into the DG area of the bilateral hippocampus of 5 × FAD + shWnt5a group and 5 × FAD + EA + shWnt5a group; the rAAV-U6-DIO-shRNA (scramble)-CMV-EGFP-SV40 polyA virus were injected into the same location of 5 × FAD + EA + shC group. Coordinates of hippocampal DG area: from Bregma AP: − 2.00 mm, ML: ± 1.3 mm, DV: − 2.3 mm. The virus was delivered using a syringe pump at a rate of 20 nl/min for 10 min, for a total of 200 nl/infusion. The syringe was then raised.2 mm, and remained in place for 10 min after each injection to allow for virus diffusion, and was then slowly retracted. Virus expression was detected using a fluorescence microscope after 21 days of virus injection.

### EA intervention

According to a previous study [27], the needle was inserted into the acupoints of Baihui (DU20) and Shenting (DU24) of the 5 × FAD + EA group, 5 × FAD + EA + shWnt5a group, and 5 × FAD + EA + shC group for 2 mm. and the electrical stimulator (G6805; SMIF, Shanghai, China) was connected, 2/20 Hz, 1 mA, 30 min/day, 5 times/week, 4 week. The WT group, 5 × FAD group, and 5 × FAD + shWnt5a group mice were fixed under the same conditions but with no EA treatment.

### BrdU injection

All mice received a single dose of 50 mg/kg BrdU (BOSTER Bio, ED1100) intraperitoneally on the 1st, 3rd, 5th …28th day of electroacupuncture (twice a day). 100 mg of BRDU powder is dissolved into a 10 mg/ml solution by physiological saline.

### OFT

The experimental scheme was set according to the reference [28], OFT was carried out in a white plastic chamber (40 × 40 × 50 cm) with a camera mounted on the top to record the behavior and activity tracking of mice (Supermaze, Softmaze, China). The chamber bottom area is divided into 25 small cells by 3 equidistant horizontal parallel lines and 3 equidistant vertical parallel lines, with the middle 9 small cells defined as the central area. The mouse was singly placed in the center and allowed to move freely to habituate the arena for 5 min and the variables recorded were total Distance, central area distance, and time spent in the central part.

### OLT

According to the reference [29], OLT consists of training sessions (10 min) and test session (5 min), separated by 1 h intervals. During the training session, we placed mice in an arena that contained two identical objects placed on the same side (A1 and A2). During the test session, object A2 was moved to another top so that A1 and A2 were placed diagonally. The mouse was placed in the chamber again and the time spent exploring two objects in 5 min was recorded as TA1 and TA2. Exploration is defined as the mouse sniffing or touching with its forepaws within 2 cm of the object. The test results are expressed by the identification index: identification index = TA2/(TA1 + TA2) × 100%.

### TUNL

The touchscreen experimental chamber consists of a touchscreen display, an infrared detector, an indoor lighting lamp, a trough tray, and a liquid dispenser. Strawberry milk was used as the liquid food reward for mice in this study (Fig. 4A). The TUNL test can only be conducted on mice after at least 3 weeks of pre-training. According to the performance of mice, 5–6 days of training should be conducted every week. With reference to the training method of Kim [30], the whole training is divided into two parts:

Pre-training stage: (1) Weight control: All mice needed food restriction and began maintaining 85–90% of free-feeding body weight throughout the experiments. (2) Environmental habituation: each mouse will explore freely for 40 min to habituate the environment. (3) Initial touch training: mice needed to complete 30 trials that touching a bright white square on the screen would result in more food rewards in 60 min. (4) Must touch training: mice need to complete 30 trials that touching will be rewarded only when white squares light up on the screen in 60 min. (5) Must initial training: the mice need to learn that the prompt light must be on at the end of the delay, and close to the food trough can trigger the random appearance of white squares. The mouse must touch 30 white squares within 45 min to receive a reward. (6) Correct touch training: The mouse must touch the white square to get a reward. If not, it will be punished by strong light. The mice completed 48 tests within 30 min for two consecutive days, and the correct rate exceeded 80% (Fig. 4B).

TUNL task: TUNL task consists of a sampling stage and a selection stage, as shown in (Fig. 4C, D). After startup, a white square appears randomly in one of the five positions on the screen. The activation was completed by blocking the infrared beam near the reward trough. After the mouse touched the white square with its nose, the stimulus was removed and began to delay. Then block the infrared beam to start the selection phase. In the selection phase, two white squares were presented, one in the wrong position in the sampling phase (wrong) and the other in the new position (correct). Touching the right position will be rewarded. The start delay (15 s) was triggered when the reward was eaten, and then the next test started. Touching a square (wrong) stimulus causes a timeout and a light penalty, after which there is a delay period before the next test. Record the total test times and the times of touching the correct position of each mouse in 45 min or the times of touching the correct position in 36 tests.

### Immunohistochemistry

Immunohistochemistry was performed with paraffin-embedded slices and immunohistochemistry kit (MXB biotechnologies, KIT-9720) to measure Aβ. Primary antibody Aβ_1-42_ (Proteintech, 25524-1-AP, 1:100) was used, and DAB chromogenic solution was added, counterstained with hematoxylin, and the slices were sealed with neutral gum. The number of Aβ plaques in the DG area of the hippocampus was counted by light microscopy. Nine brain slices (three mice, three consecutive slices per mouse) from each group were taken for counting the Aβ plaque number of DG area in a microscopic field of × 100.

### Immunofluorescence

Immunofluorescence used paraffin-embedded sections with a thickness of 4 μm. And 0.3% Triton was added to the brain slices to permeabilize the cell membrane for 25 min. The DNA was denatured with hydrochloric acid at 37 °C for 1 h, after which the tissue was covered with 5% fetal bovine serum plus 5% goat serum blocking solution and incubated at 37 °C for 1 h. Then, primary antibodies, BrdU (Abcam, ab8955, 1:100) and CaR (Cell Signaling Technology, CST92635, 1:100), were used over at night 4 °C. Goat anti-Mouse 555 (Abcam, ab150114, 1:500), Goat anti-Rabbit 633 (Thermo Fisher, A21070, 1:500) were used as second antibodies, and nuclei were visualized with DAPI. Cells expressing both BrdU and CaR were defined as new immature GCs. The number of BrdU^+^/CaR^+^ cells in the hippocampal DG was counted by laser scanning confocal microscopy.

### Golgi staining

The Golgi staining was performed with the Golgi staining kit (Fdneurotech, PK401A). The soaked brain tissue was embedded in a frozen section embedding agent (100 um). The slides were dried at room temperature in the dark for at least 12 h. Subsequently, the slides were washed twice in double steamed water for 4 min each time. After that, they were successively dehydrated in 50%, 75%, and 90% ethanol for 4 min each time. Each gradient of ethanol was dehydrated and then placed into absolute ethanol for further dehydration, 4 times for 4 min each time. After completion of dehydration, the cells were placed in xylene 3 times for 4 min each for transparency. The stained slides were sealed and stored in the dark. Dendrites in the DG were photographed under a microscope at a magnification of × 630.

### Polymerase chain reaction

Total RNA was extracted from brain tissues using TRIzol reagent (Vazyme Biotech Co., Ltd) and converted to cDNA using the HiScript^®^ II RT SuperMix for qPCR (+ gDNA wiper) kit (Vazyme Biotech Co., Ltd. R223). qPCR was performed with ChamQ Universal SYBR qPCR Master Mix (Vazyme Biotech Co., Ltd. Q711). All primers were purchased from SunYa biological company, Wnt5a-F(GGAACGAATCCACGATAAGG), Wnt5a-R(CAGACACTCCATGACACTTACAG). GAPDH-F(TGGAAAGCTGTGGCGTGATG), GAPDH-R(TACTTGGCAGGTTTCTCCAGG). Relative mRNA levels were calculated by normalization to the level of GAPDH. Relative gene expression was analyzed based on the fold change (the 2^−ΔΔCt^ method).

### Western blot

Taken the mouse hippocampus and adjusted the concentration to 5 μg/μl after BCA quantification. Prepared 10% SDS-PAGE gel, electrophoresis, membrane transfer, and sealed 5–8% milk for 2 h. According to the instructions, incubate PVDF membrane in the primary antibody solution (GAPDH, Proteintech, 60004–1-lg, 1:5000; CaR, Cell Signaling Technology, CST92635, 1:1000; Prox1, Abcam, ab199359, 1:1000; Wnt5a, Abcam, ab229200, 1:1000; DVL2, 12037-1-AP, 1:500; p-DVL2, ybio, YB73041,1:500; FZD2, Proteintech, 24272-1-AP, 1:1500; CaMKII,Abcam, ab52476, 1:1000; p-CaMKII,Abcam, ab32678, 1:1000) over at night 4 ℃. secondary antibodies (goat anti-rabbit, Proteintech, SA00001-2, 1:5000; Proteintech, goat anti-mouse, SA00001-1, 1:5000) at room temperature for 1 h. Use developer to cover PVDF film, develop in Bio-Rad imager, use Image lab software to calculate protein integrated density, and then put it into SPSS for statistical analysis.

### Statistical analysis

All test data in this study were analyzed in SPSS25.0 statistical software. If the measurement data conform to the normal distribution, the experimental data shall be expressed as mean ± standard deviation. One-way ANOVA was used for comparison between data groups. The LSD method was used to compare the two groups when the variance was homogeneous. When the conflict is homogeneous, the Games Howells method compares two groups. *P* < 0.05 was considered statistically significant.

## Results

1. 3-month-old 5 × FAD mice showed impaired pattern separation, and EA may exert beneficial effects through Wnt5a

**Fig. 1:**
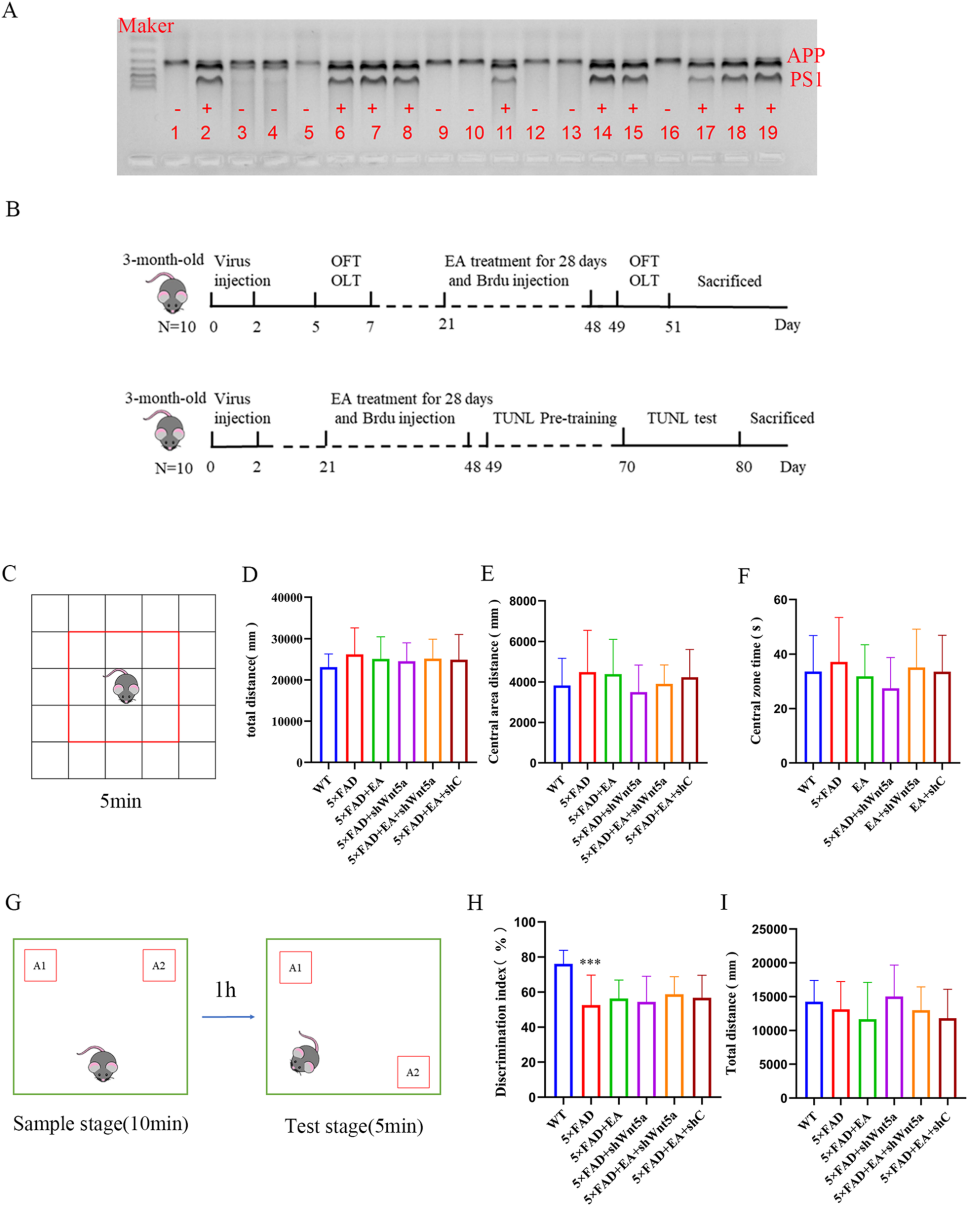
Experimental design and preintervention cognitive behavioral testing (**A**) Gene identification results. The maker brand from top to bottom represent 100, 200, 300, 400, 500, 600, 700 bp. APP = 377 bp, PS1 = 608 bp, GAPDH = 324 bp. Mice numbered 2, 6, 7, 8, 11, 14, 15, 17, 18, and 19 in the figure represent AD positive. **B** experimental design. **(C)** OFT schematic. The middle nine squares were defined as the central region. The results of OFT before EA intervention, there were no differences in total movement distance (**D**) and central area distance (**E**) or central area time (**F**) among the groups, indicating that the overall exploration ability and emotional state of the mice were normal. **G** Schematic representation of the OLT. After 10 min of free exploration, a 5-min test was performed one hour later. A1 and A2 are two objects that look the same. The OLT results before intervention showed decreased memory discrimination in AD mice compared with wild-type mice (**H**), indicating impaired pattern separation. The motor ability of each mouse was consistent (**I**). All data are presented as mean ± SD; N = 10 mice; ^***^P < 0.001 vs. WT group;

After completion of gene identification (Fig. 1A), we carried out the experiments according to the schematic representation (Fig. 1B). Firstly, a series of behavioral tests were conducted to identify cognitive impairment at the early stage of AD. OFT results before intervention showed no statistical difference in each group’s total distance (Fig. 1C, D), central area distance (Fig. 1E), and central area time of the OFT (Fig. 1F). This observation suggests indicates that the exploration and exercise ability of mice in each group were consistent, indicating an absence of anxiety-like behavior.

The OLT results before intervention revealed a significant decrease in the discrimination index of the other five groups of mice compared to the wild-type group (Fig. 1G, H), indicating impaired memory discrimination in 3-month-old mice. The total exercise distance did not differ significantly among the groups (Fig. 1I), meaning that the decline of the discrimination index was unrelated to exercise ability.

**Fig. 2:**
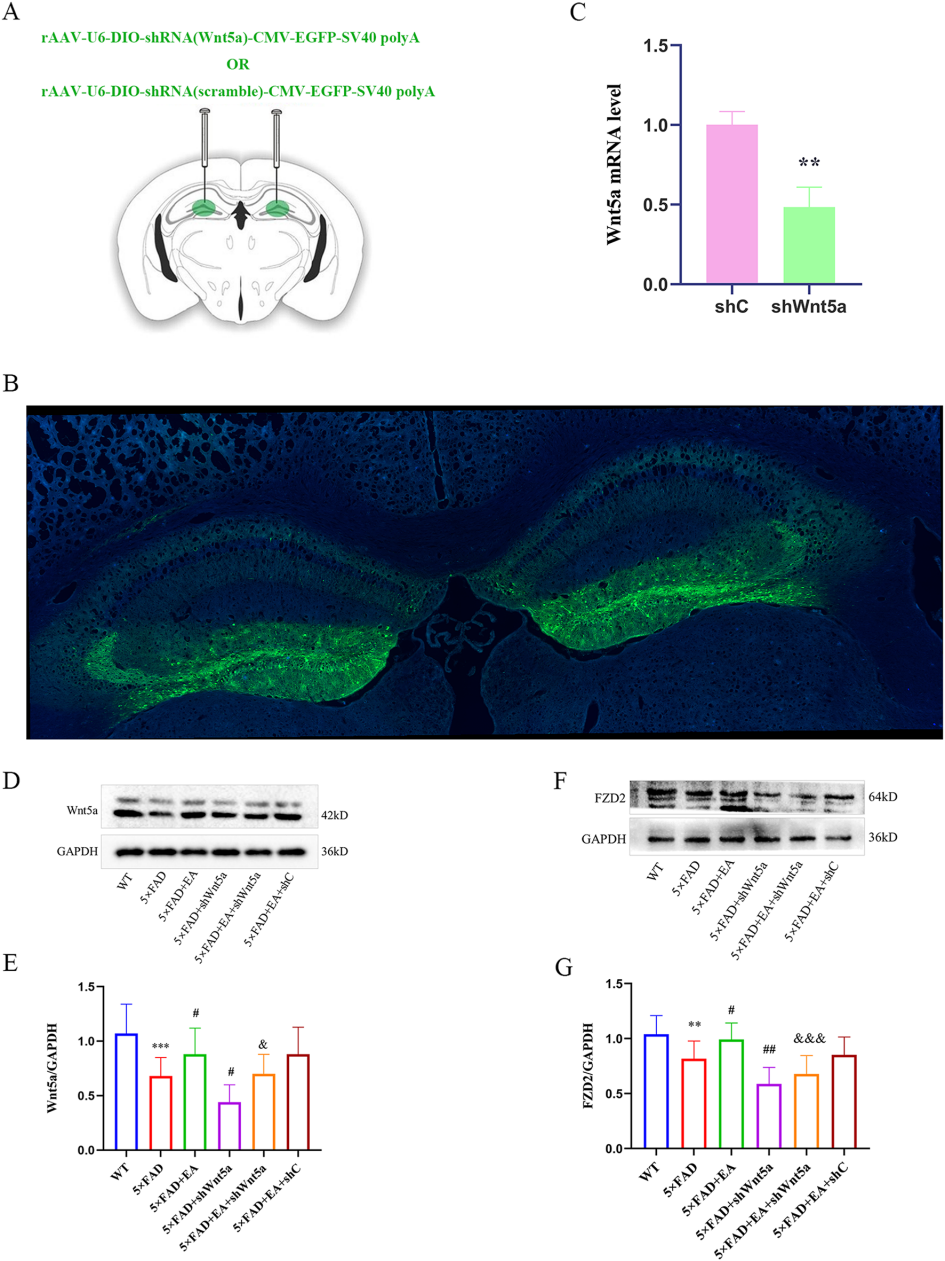
Knockdown of Wnt5a using rAAV and validation of knockdown efficiency (**A**) shWnt5a or shC injected into the DG bilaterally. Coordinates of hippocampal DG area: from Bregma AP: − 2.00 mm, ML: ± 1.3 mm, DV: − 2.3 mm (**B**) Viral expression map of the DG. **C** Wnt5a mRNA levels were detected by qPCR after virus injection. (**D**, **E**) Western blot pictures and statistical results of Wnt5a expression levels in the hippocampus of mice in each group; (**F**, **G**) Western blot pictures and statistical results of FZD2 expression levels in the hippocampus of mice in each group. All data are presented as mean ± SD; N = 3 mice; ^***^P < 0.001 vs. WT group; ^#^*P* < 0.05 vs. 5 × FAD group; ^&^*P* < 0.05, ^&&&^*P* < 0.001 vs EA group

To evaluate the role of the Wnt5a in EA promoting cognitive, Wnt5a was knocked down by recombination adeno-associated virus (rAAV)-mediated shRNA. In these experiments rAAV vectors co-expressing mouse Wnt5a-targeting shRNA (shWnt5a) or control shRNA (shC) and enhanced EGFP were used in hippocampal DG (Fig. 2A). Firstly, we examined the accuracy of rAAV injection location and could clearly see EGFP expression in the DG region (Fig. 2B). Secondly, the efficiency of Wnt5a silencing was examined using qPCR. A significant reduction in Wnt5a mRNA levels was observed in the DG region of mice injected with shWnt5a compared to mice injected with shC (Fig. 2C).

After 4 weeks of EA, we examined Wnt5a expression levels in the hippocampus of mice from each group (Fig. 2D, E). We can see the expression of Wnt5a in the 5 × FAD group was lower than in the WT group, which suggests that under AD pathology, Wnt5a expression levels are decreased. Compared with the 5 × FAD group, the expression of Wnt5a in the 5 × FAD + shWnt5a group decreased, which indicated that shWnt5a exerted the effect of knocking down the expression level of Wnt5a. Compared with the 5 × FAD group, the expression of Wnt5a in the 5 × FAD + EA group increased. Compared with the 5 × FAD + EA group, the expression level of Wnt5a in the 5 × FAD + EA + shWnt5a group decreased, and there was no difference in the 5 × FAD + EA + shC group (Fig. 2E). These results indicate that Wnt5a content is reduced under pathological conditions and electroacupuncture can promote Wnt5a expression. Furthermore, Frizzled2(FZD2) is a strong binding receptor with Wnt5a, and we examined FZD2 expression levels in the hippocampus of mice from each group (Fig. 2F, G). This result is consistent with the level of Wnt5a expression and suggests that the level of Wnt5a expression can cause changes in FZD2 compliance.

**Fig. 3:**
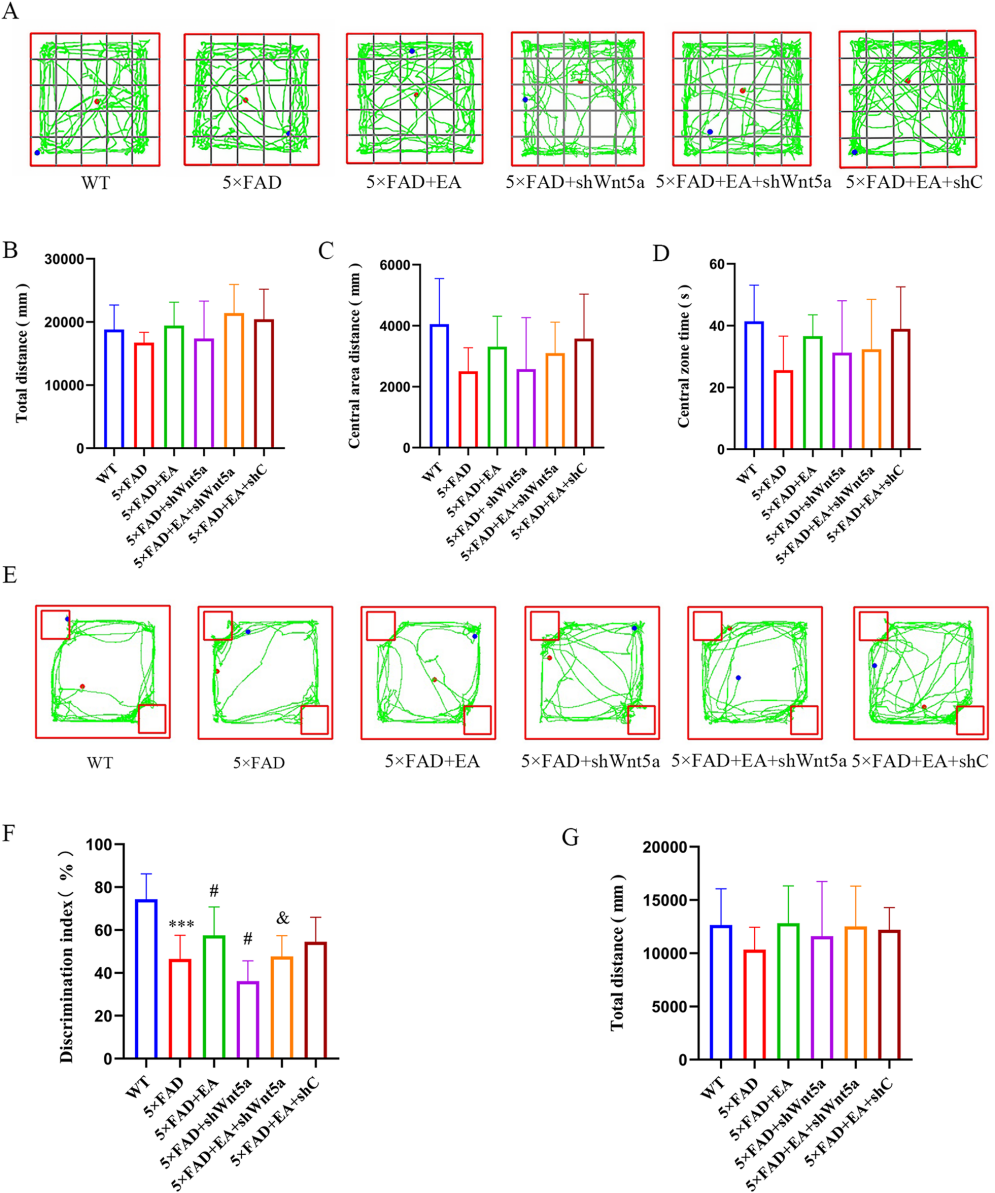
OFT and OLT performance of mice in each group after EA intervention and virus injections. **A** Representative plots of OFT trajectories in each group of mice after EA intervention and virus injections. There were no differences in total distance (**B**), the central area distance (**C**) and the central area time (**D**) among each group, indicating that the overall exploration ability and emotional state of the mice were normal. **E** Representative plots of OLT trajectories in each group of mice after virus injection and EA intervention. EA could improve the memory discrimination ability. Interference of Wnt5a expression aggravated the impairment of memory discrimination in AD mice, and the effect of EA was reduced (**F**). The motor ability of each mouse was consistent (**G**). All data are presented as mean ± SD; N = 10 mice; ^***^P < 0.001 vs. WT group; ^#^*P* < 0.05 vs. 5 × FAD group; ^&^*P* < 0.05 vs EA group

After 4 weeks of EA, cognitive behavioral tests were executed again in each group. The OFT results showed that there was no statistical difference in the total distance (Fig. 3A, B), central area distance (Fig. 3C), and central area time of the OFT in each group (Fig. 3D), which means that the exploration ability of mice in each group was consistent after EA, and EA did not cause anxiety in mice. The OLT results showed that the 5 × FAD group's discrimination index decreased compared with the WT group (Fig. 3E, F). Compared with the 5 × FAD group, the discrimination index of the 5 × FAD + EA group increased, and the discrimination index of the 5 × FAD + shWnt5a group decreased. Compared with the 5 × FAD + EA group, the discrimination index of the 5 × FAD + EA + shWnt5a group decreased, and the 5 × FAD + EA + shC group had no difference. There is no statistical difference in the total distance of each group (Fig. 3G). It shows that EA can improve the memory discrimination ability of AD mice, and Wnt5a participates in the process of EA improving the memory discrimination ability of AD mice.

**Fig. 4:**
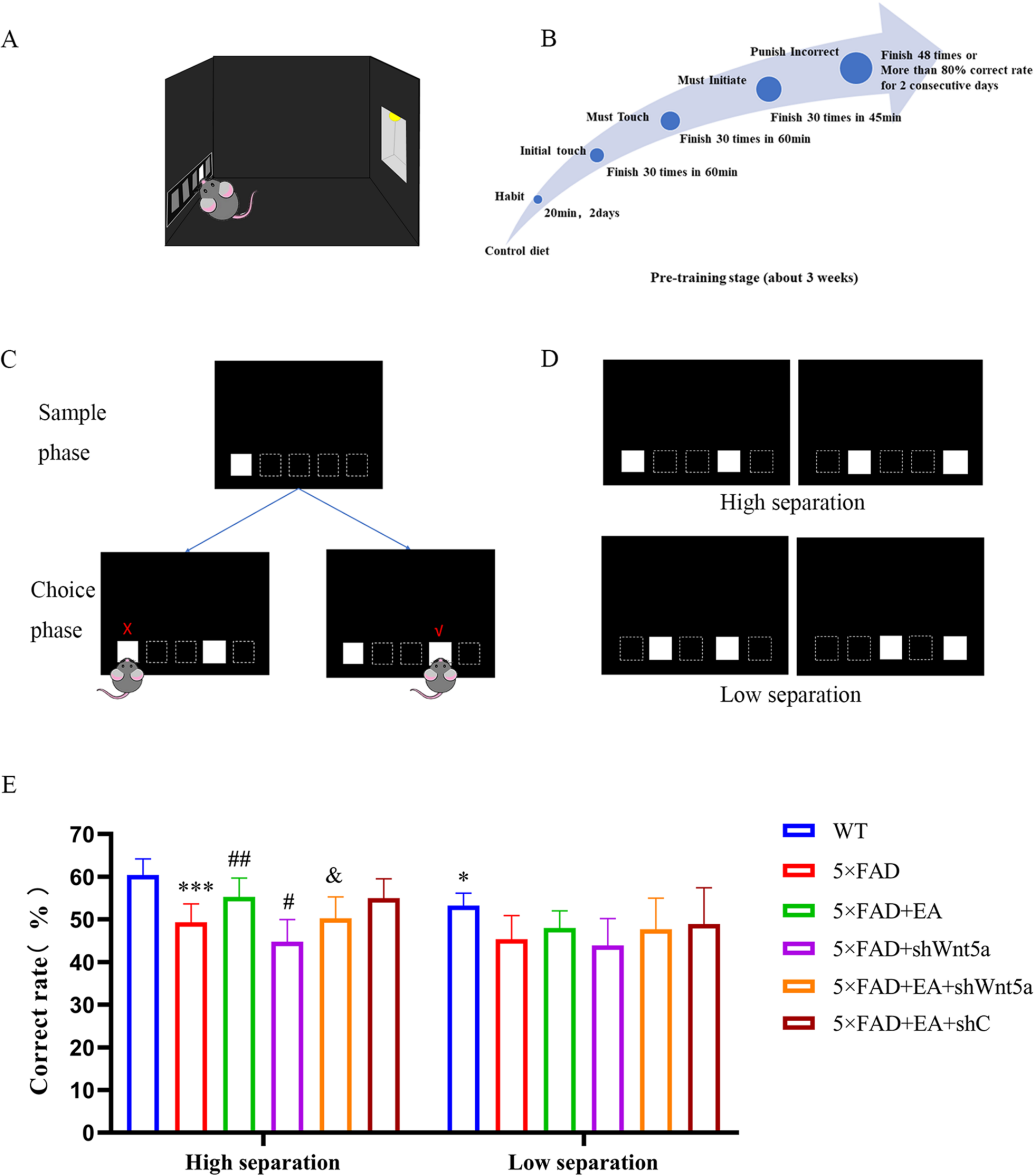
Assessment of pattern separation ability (**A**) Model separation test chamber simulation diagram (**B**) The learning process and the time required before the TUNL test. **C** TUNL test design. TUNL was divided into sampling and selection phases. The mouse's selection of the newly presented square during the choice phase was counted as correct. **D** Two kinds of experiments were designed in the selection phase. The smaller the separation interval, the harder it is to distinguish and the higher the requirement for pattern separation ability. **E** TUNL accuracy. At the high separation level, AD mice showed impaired pattern separation. EA improved pattern separation ability, interfered with Wnt5a expression, aggravated pattern separation ability, and the effect of EA was weakened. At the low separation, the pattern separation of AD mice decreased, and EA and interference with wnt5a did not affect the pattern separation ability. That is, the pattern separation ability reached the upper limit. All data are presented as mean ± SD; N = 10 mice; ^*^P < 0.05 and ^***^P < 0.001 vs. WT group; ^##^*P* < 0.01 vs. 5 × FAD group; ^&^*P* < 0.05 vs EA group

Pattern separation mediates the discrimination of similar memories. We used the TUNL task to detect the pattern separation ability of mice in each group after intervention (Fig. 4A–D). The results showed that: Under the difficulty of high separation interval (Fig. 4E), compared with the WT group, the correct rate of the 5 × FAD group decreased. The 5 × FAD + EA group had a higher accuracy rate than the 5 × FAD group, and the 5 × FAD + shWnt5a group had a lower accuracy rate. Compared with the 5 × FAD + EA group, the correct rate in the 5 × FAD + EA + shWnt5a group was decreased, and there was no difference in the 5 × FAD + EA + shC group. These results indicate that EA can improve the ability of pattern separation in AD mice, and Wnt5a is involved in this effect. Under low separation difficulty (Fig. 4E), only the 5 × FAD group had a lower accuracy rate than the wild-type group, and there was no difference in the accuracy rate of the other groups, indicating that the effect of EA on improving pattern separation was no longer obvious when the difficulty was increased.

2. EA decreased Aβ plaque deposition in the hippocampus DG area of 5 × FAD mice

**Fig. 5:**
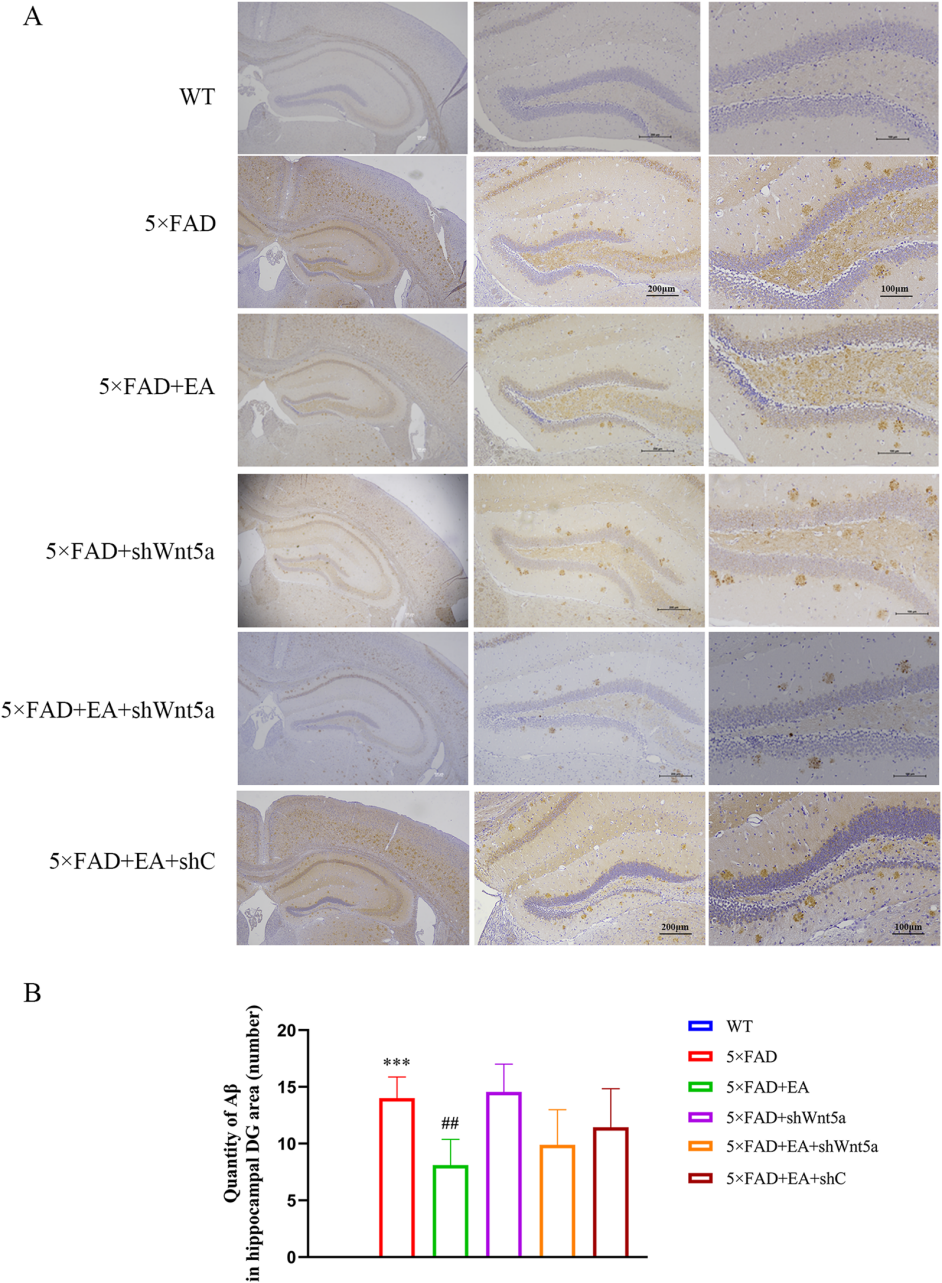
Aβ plaque deposition counts in the DG area (**A**) Immunohistochemical representative plots of Aβ in the DG region of mice in each group. The magnification from left to right is 40x, 100x, 200x. **B** There were many Aβ plaques in the DG region of AD mice, and EA could reduce the deposition of Aβ plaques. But interference with Wnt5a expression did not affect the formation of Aβ plaques and did not inhibit the effect of EA. All data are presented as mean ± SD; n = 3 mice; ^***^P < 0.001 vs. WT group; ^##^*P* < 0.01 vs. 5 × FAD group

To clarify the reasons for the decline of memory discrimination ability, we measured the levels of Aβ_1-42_ in the DG area. The results showed that there was no Aβ plaque deposition in the hippocampal DG area in the wild-type group, but the number of Aβ plaques increased in the 5 × FAD group. Compared with the 5 × FAD group, the number of plaques in the 5 × FAD + EA group was reduced, and there was no difference in the number of plaques in the 5 × FAD + shWnt5a group. Compared with the 5 × FAD + EA group, there was no significant difference in the number of plaques in the 5 × FAD + EA + shWnt5a group and the 5 × FAD + EA + shC group. These results indicated that EA inhibited Aβ deposition, and Wnt5a had no effect on the formation of Aβ (Fig. 5A, B).

3. The neurogenesis of immature GCs can be promoted by EA in the hippocampal DG area, and Wnt5a participates in the process

A previous review article extensively discussed the role of Prox1 in neuronal differentiation within the DG area and its significance as a key factor for granule cell maturation [31]. The findings of previous studies have demonstrated that the overexpression of Prox1 facilitates the differentiation process of neural stem cells into granule cells, whereas inhibition of Prox1 expression hampers neuronal differentiation [32]. Therefore, we assess neural differentiation levels in the DG region among different groups by evaluating Prox1 expression levels. In addition, BrdU, a reagent capable of substituting thymidine in the DNA synthesis phase (S phase) and specifically reacting with Apollo fluorescent dye to detect cell proliferation, was intraperitoneally injected into mice during EA to further elucidate the impact of EA on neurogenesis in the DG region. To ascertain whether the proliferating cells were immature granule cells, double staining of car, an immature granule cell marker [33], and Brdu was conducted.

**Fig. 6:**
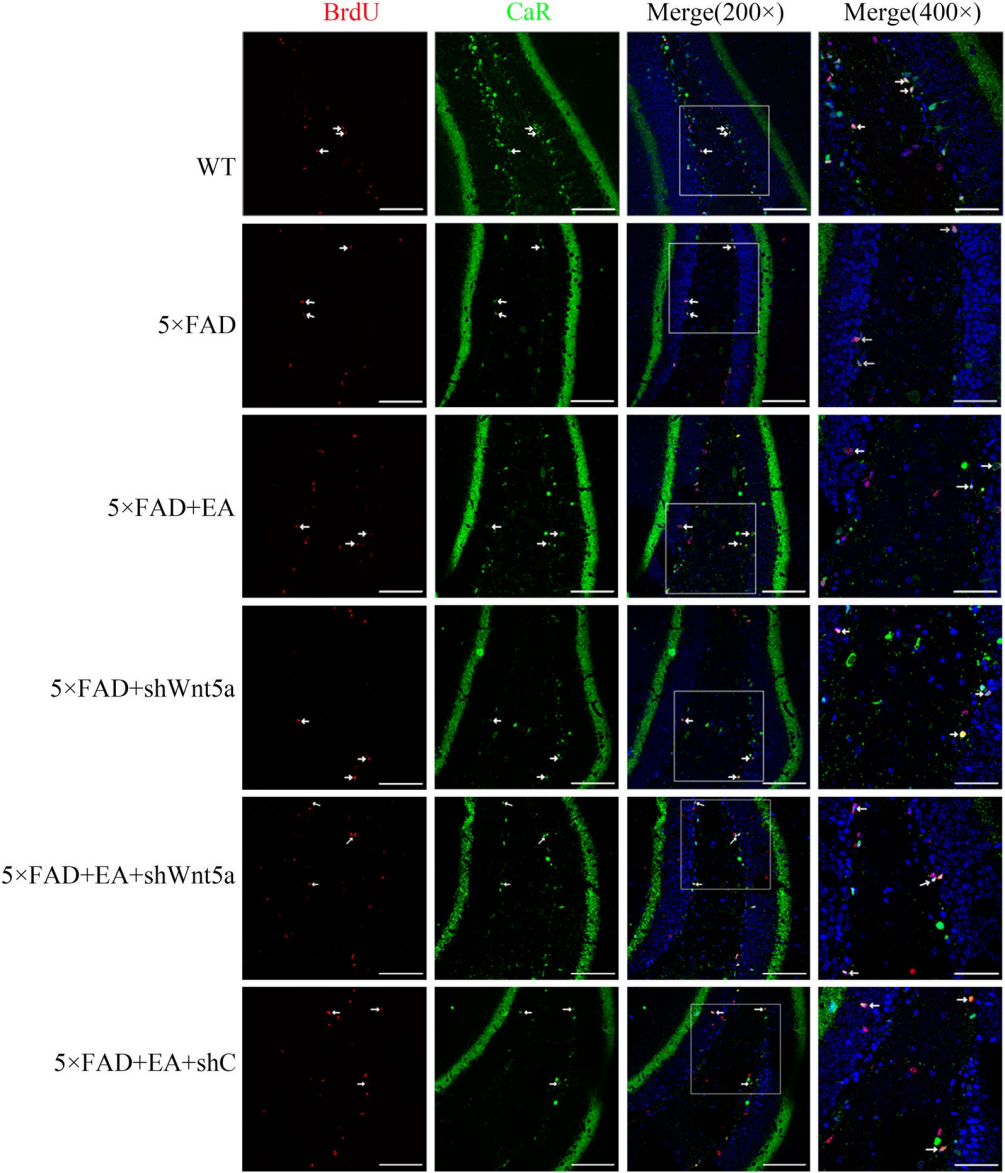
Representative 200X, 400X photomicrographs of immature GCs in the DG area Newborn neurons were labeled with BrdU, and GCs were labeled with CaR protein. Neurons that also expressed BrdU and CaR were newly immature GCs

**Fig. 7:**
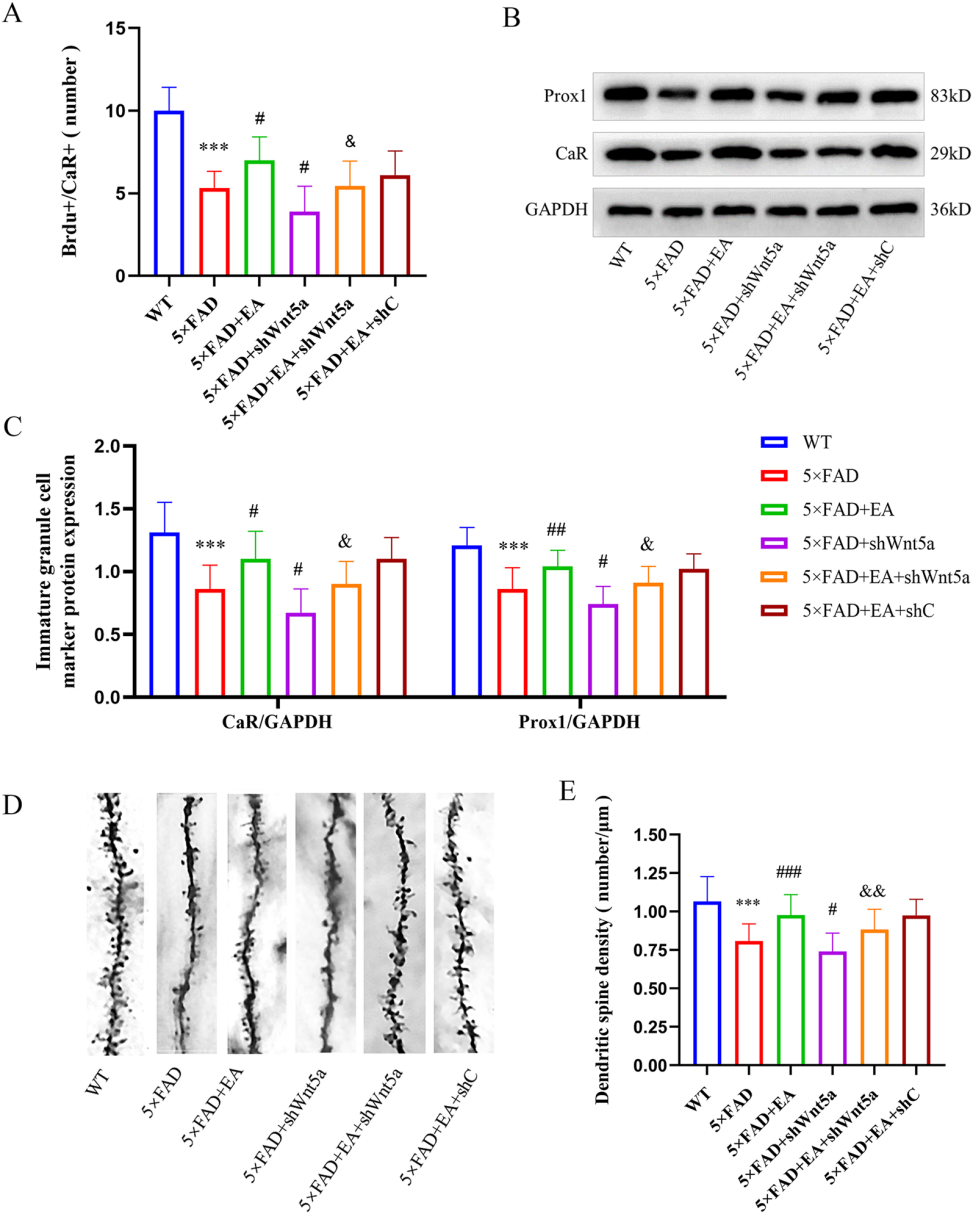
**A**. The cell number of BrdU^+^/CaR^+^ (**B**, **C**) Western blot was used to detect the level of neurogenesis, and the results of Western blot were consistent with the fluorescence results. (**D, E**) The developmental level of neuronal dendrites and 630X photomicrographs of neuronal dendrites in DG. All data are presented as mean ± SD; n = 3 mice; ^***^P < 0.001 vs. WT group; ^#^*P* < 0.05 and ^##^*P* < 0.01 vs. 5 × FAD group; ^&^*P* < 0.05 vs EA group

The results showed that the number of BrdU^+^/CaR^+^ cells in the 5 × FAD group decreased compared with the WT group. Compared with the 5 × FAD group, the number of BrdU^+^/CaR^+^ cells in the 5 × FAD + EA group increased, and the 5 × FAD + shWnt5a group was decreased. Compared with the 5 × FAD + EA group, the number of BrdU^+^/CaR^+^ cells was decreased in the 5 × FAD + EA + shWnt5a interfering virus group, and there was no difference in the 5 × FAD + EA + shC group(Figs. 6, 7A); It shows that the neurogenesis ability of immature GCs in AD model mice decreased. EA can promote the neurogenesis of immature GCs, and Wnt5a is involved in the process.

In addition, Western blot showed that compared with the WT, the expression of CaR and Prox1 in the 5 × FAD group decreased (Fig. 7B). Compared with the 5 × FAD group, the expression of CaR and Prox1 increased in the 5 × FAD + EA group, while the expression of CaR and Prox1 decreased in the 5 × FAD + shWnt5a group. Compared with the 5 × FAD + EA group, the expression of CaR and Prox1 decreased in the 5 × FAD + EA + shWnt5a group, and there was no difference in the 5 × FAD + EA + shC group (Fig. 7C); These results indicated that EA could promote the neurogenesis of immature GCs, and Wnt5a participates in the process.

4. EA improves the density of dendritic spines in the hippocampus DG

Synapses are formed between neurons through dendritic spines to complete information transmission. We assessed neuronal dendritic spine density in the hippocampus of each group to indirectly reflect the level of neurogenesis (Fig. 7D). Compared with the WT group, the density of dendritic spines in the 5 × FAD group decreased. Compared with the 5 × FAD group, the density of dendritic spines in the 5 × FAD + EA group increased, and the density of dendritic spines in the 5 × FAD + shWnt5a group decreased. Compared with the 5 × FAD + EA group, the density of dendritic spines of the 5 × FAD + EA + shWnt5a group decreased, and there was no difference between the 5 × FAD + EA + shC group (Fig. 7E). The results indicate that the development of neuronal dendritic spines is abnormal in 5 × FAD model mice, and EA can promote the formation of dendritic spines. The results suggest that the development of neuronal dendritic spines is abnormal in 5 × FAD model mice, and EA can promote the formation of dendritic spines.

5. EA can activate the Wnt signaling pathway by regulating Wnt5a in the DG area of 5 × FAD mice

**Fig. 8:**
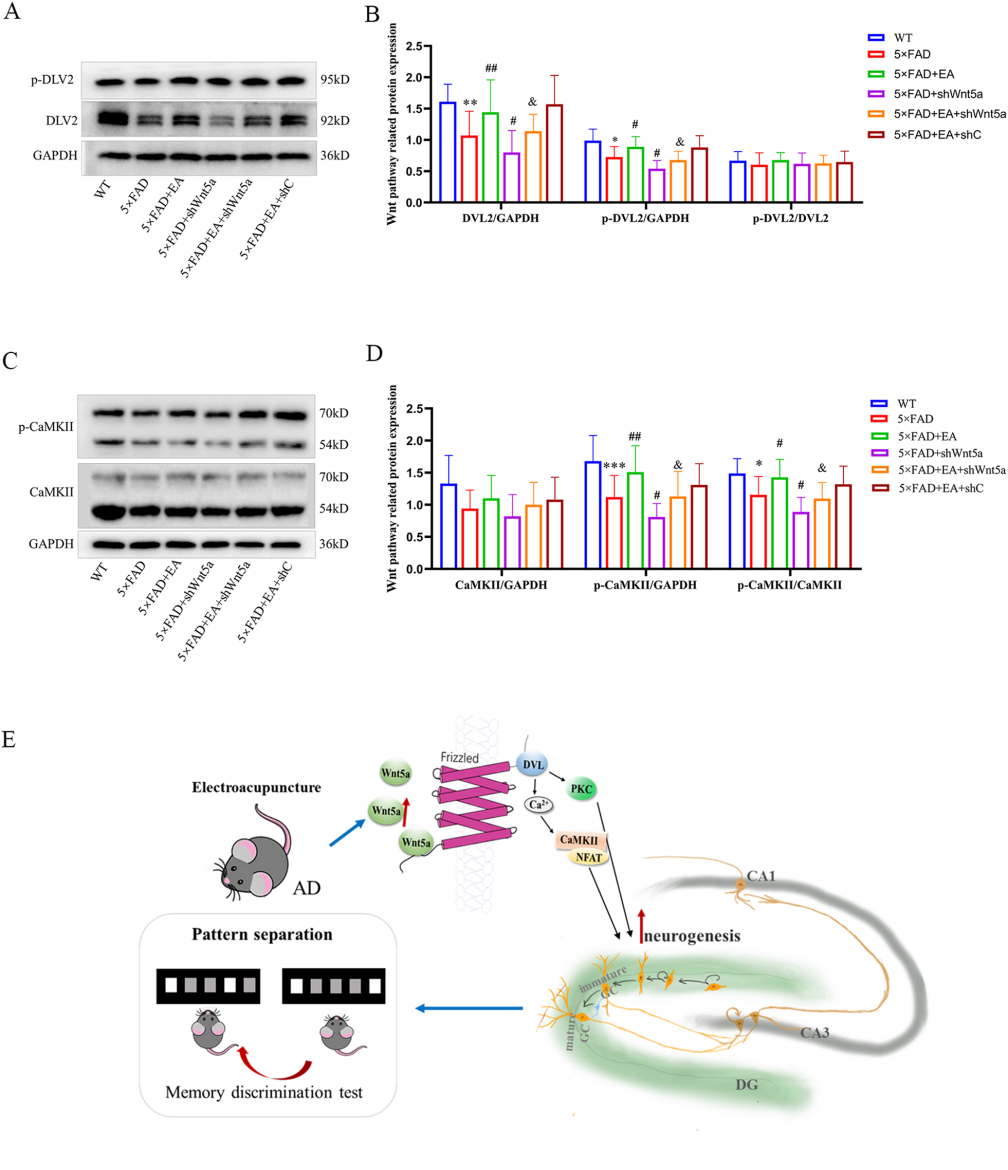
the expression levels of Wnt signaling pathway-related proteins (**A**, **B**) The expression level of total DVL2 and p-DVL2; (**C**, **D**) The expression level of total CaMKII and p-CaMKII. All data are presented as mean ± SD; n = 3 mice; ^**^P < 0.01 and ^***^P < 0.001 vs. WT group; ^#^*P* < 0.05 and ^##^*P* < 0.01 vs. 5 × FAD group; ^&^*P* < 0.05 vs EA group; (**E**) Schematic representation of the study conclusions

To further reveal how EA promotes neurogenesis, we examined an essential signaling pathway in neurogenesis, the Wnt signaling pathway. DVL2 is one of the Wnt5a receptors within the cell membrane. The expression of p-DVL2 and total DVL2 in the 5 × FAD group decreased compared with the WT group. Compared with the 5 × FAD group, the expression level of p-DVL2 and total DVL2 in the 5 × FAD + EA group increased, while that in the 5 × FAD + shWnt5a group decreased. Compared with the 5 × FAD + EA group, the expression level of p-DVL2 and total DVL2 of the 5 × FAD + EA + shWnt5a decreased, and there was no difference in the 5 × FAD + EA + shC group. But there was no significant difference in the expression of p-DVl2 /DVL2 among groups (p > 0.05). (Fig. 8A, B). At the expression level of p-CaMKII and p-CaMKII/CaMKII, compared with the WT group, the expression of p-CaMKII and p-CaMKII/CaMKII in the 5 × FAD group decreased; Compared with the 5 × FAD group, the expression level of p-CaMKII and p-CaMKII/CaMKII in the 5 × FAD + EA group increased, while that in the 5 × FAD + shWnt5a group decreased; Compared with the 5 × FAD + EA group, the expression level of p-CaMKII of 5 × FAD + EA + shWnt5a decreased, and there was no difference in the 5 × FAD + EA + shC group. There was no significant difference in the expression of total CaMKII among groups. (Fig. 8C, D). The results indicate that the activation of Wnt signaling pathway is decreased in AD model mice, and EA can activate Wnt signaling pathway.

## Discussion

Our results showed that 3-month-old 5 × FAD mice exhibited impaired spatial memory discrimination and reduced neurogenesis in the hippocampal DG region, with Wnt5a playing a crucial role in regulating neurogenesis and pattern separation ability in mice. EA can effectively promote neurogenesis in the DG and enhance pattern separation ability in early AD model mice, potentially through modulation Wnt5a expression levels. The overall experimental results are shown in the diagram (Fig. 8E).

1. Impairment of pattern separation leads to the decline of AD memory discrimination

Cognitive decline, particularly memory, is a pivotal clinical manifestation observed in patients with AD. In the pattern separation task, compared with normal adults, patients with MCI and AD showed a decline in pattern separation ability, and patients with AD had the weakest ability [5–7]. Previous studies have demonstrated that 3-month-old 5 × FAD mice exhibit impairments in spatial location discrimination rather than object discrimination [27]. The objects position discrimination test was conducted in this study to assess the memory discrimination ability of 3-month-old model mice. The findings revealed spatial memory discrimination impairment in mice at this age, which aligns with previous research results.

The TUNL task is a behavioral method available in rodents that can assess working memory and pattern separation. This method provides an accurate assessment of animals' autonomous learning and discrimination capabilities while minimizing potential confounding factors associated with human intervention [34–36]. In the TUNL task, we set two test difficulties of high separation and low separation. Our results showed that the pattern separation ability of mice is inherently limited, as evidenced by a decline in memory discrimination accuracy with increasing difficulty. When the difficulty exceeded the maximum limit of pattern separation ability, the disease did not affect the discrimination performance. Lauren also observed this phenomenon in mice with impaired neurogenesis and mice with damage to the dorsal hippocampus. The difference is that they also performed TUNL tests with more significant separation and found no difference in memory discrimination between wild-type mice and 5 × FAD mice when the separation interval was increased [30, 37].

The hippocampal DG region is among the brain areas that retain the capacity for neurogenesis in adulthood. In animal studies, performance on the pattern separation task has been found to correlate with the level of neurogenesis in the DG, and animals exhibiting impaired neurogenesis in this region have demonstrated deficits on the hippocampus-dependent recognition tasks [38–40]. Neurogenesis in the DG area gives rise to immature GCs that can be integrated into the classic trisynaptic circuit [41]. Previous studies have found that newborn immature GCs are involved in pattern separation, whereas mature GCs are responsible for pattern completion [42]. Shay found that the number of immature GCs, LTP in the DG region, and the ability to distinguish similar situations were reduced by using X-rays to inhibit neurogenesis in the DG region [16]. When using lentivirus to inhibit the Wnt signaling pathway, Clelland found a significant decline in the number of DCX^+^ cells in the hippocampal DG region, along with a decrease in the accuracy of the touchscreen pattern separation test [40]. According to the studies of Kronenberg et al., the hallmark of the early stages of adult nascent granulosa cell development is CaR expression, which lasts for about 3 weeks and is subsequently converted to calcium-binding protein [1], so we finally identified CaR as the marker proteins of immature GCs. Prospero homeobox 1 (Prox1), a transcription factor expressed in the granule cell lineage, is expressed in granule cells at all stages of DG development and in adult-born granule cells. It is required for the maturation of granule cells in the DG region during development and for the maintenance of an intermediate progenitor state during adult neurogenesis [43]. Therefore, our study aims to assess neural differentiation levels in the DG region among different groups by evaluating Prox1 expression levels. And the quantification of immature granule cells and the expression level of marker proteins were conducted to assess neurogenesis in the DG region of the hippocampus. Our findings demonstrate that 5 × FAD mice impaired neurogenesis, potentially contributing to their deficits in memory discrimination.

2. Wnt5a is an essential protein involved in neurogenesis within the DG region and exerts an impact on the capacity for pattern separation

In the central nervous system, the Wnt signaling pathway controls key processes such as cell division, differentiation, polarity, migration, and synaptogenesis [44–48]. In the signaling process, Wnt proteins bind to the Frizzled (FZD) and LRP5/6 receptor complex, inducing a conformational change in the receptor complex that subsequently activates downstream pathways [49]. It was found that the expression levels of Wnt signaling pathway genes in the medial temporal lobe of AD patients changed, and the hyperphosphorylation of GSK-3β in the pathway co-occurred with tau aggregation in insoluble neurofibrillary tangles in the brain of AD patients [50]. In addition, genetic variations in the Wnt coreceptor LRP6 lead to reduced Wnt signaling activation, which is associated with late-onset AD [51]. Wnt5a is a neurogenic factor that mediates neuronal differentiation through Wnt/Ca^2+^/CaMKII signaling and neuronal morphological development through Wnt/Ca^2+^ and Wnt/JNK cascades [23]. In the present study, we observed a reduction in both Wnt5a and its major receptor, FZD2, in the hippocampus of AD mice. Studies have demonstrated a significant upregulation in the expression of Wnt signaling inhibitor proteins (such as DKK-1, SFRP-1, and SFRP-2) in postmortem AD brain and transgenic AD mouse models [52, 53].This upregulation effectively suppresses the Wnt signaling pathway in Alzheimer's disease (AD). Consequently, there is a subsequent decrease observed in the expression levels of WnT-related proteins (e.g., wnt5a, wnt3a) and various intracellular related proteins (e.g., FZD2, DVL2, GSK3β). Notably, DKK-1 has been shown to interact with LRP5/6 to mediate Aβ-induced inhibition of Wnt signaling [54]. Additionally, secreted frizzled-related protein (sFRP), possessing a cysteine-rich domain (CRD) similar to frizzled-related receptors, can directly bind to Wnt protein leading to a significant reduction in Wnt5a binding with FZD2 receptors on cell surfaces [55]. This may consequently result in an adaptive reduction of FZD2 expression.

In adult hippocampal progenitor cells, Wnt5a expression is essential for the differentiation and the morphological development of newborn neurons, which is related to the activation of CaMKII, PKC, and JNK by Wnt5a [23, 56]. Wnt5a, which is expressed both pre-synaptic and post-synaptic, is an important protein that constitutes synaptic connections between neurons and affects synaptic plasticity. Knockout of Wnt5a leads to impaired LTP and decreased recognition memory ability in mice [20]. In this study, we found that Wnt5a expression was reduced in AD mice, so we speculated that Wnt5a was involved in neurogenesis and affected pattern separation performance. To further verify this hypothesis, we used a virus to knock down Wnt5a expression and found that the number of newly born immature GCs was decreased in the DG region of the hippocampus, as was the density of neuronal dendritic spines, which was consistent with the results of previous studies [23]. At the same time, the discrimination index of OLT and the accuracy of TUNL task decreased. Therefore, we identified an important role for Wnt5a in pattern separation. In addition, the Wnt signaling plays a vital role in the ecological development of neurons in addition to regulating neurogenesis in the DG area. We detected the density of dendritic spines of neurons in the DG area, and found that knockdown of Wnt5a expression reduced the density of dendritic spines of neurons in the DG area of mice, and the density of dendritic spines increased after EA. However, due to the lack of neuron specificity of Golgi staining, we cannot determine whether the neurons with changes in dendritic spine density are newborn immature GCs, which needs further study.

3. EA can promote immature GC regeneration and improve pattern separation by regulating the expression of Wnt5a protein

Acupuncture can serve as a complementary therapy for AD. Meta-analysis findings demonstrate acupuncture exhibits the potential to ameliorate cognitive dysfunction in AD patients, as evidenced by improvements observed in MMSE, ADL, and ADAS scores [57, 58]. Kim found that acupuncture could reduce the ADAS-cog score of patients with MCI, and the efficacy of acupuncture at 30 min was better than that at 20 min, and the effect lasted up to 3 months [59]. Previous studies have shown the effect of EA on improving cognitive dysfunction in AD mice [27, 60]. In this study, the same intervention method was used, and the results showed that acupuncture exhibited a certain degree of efficacy in enhancing memory discrimination behavior among AD mice. Still, for the difficult test, the improvement effect of EA was not obvious. At the same time, we also carried out OFT after EA, which showed that EA did not cause emotional disturbance in mice, thus excluding the influence of emotional problems on memory discrimination impairment in mice. The effect of EA on promoting neurogenesis has been observed in other disease models [24, 25, 61, 62]. Combined with our experimental results, we found that the number of newborn immature GCs in the hippocampal DG region of AD mice treated with EA was greater than that of non-EA mice, and the pattern separation ability of the mice was also improved. Therefore, we believe that EA can be used as a complementary therapy for early pattern separation disorders in AD.

Further, we tried to find the molecular mechanism by which EA improves pattern separation. We observed that EA increased Wnt5a expression in the hippocampus, and since the role of Wnt5a in improving pattern separation has been found, we hypothesized that EA might act by regulating Wnt5a expression. After interfering with wnt5a expression, we detected that the effect of EA on promoting hippocampal DG neurogenesis weakened, and the mice's pattern separation behavior test decreased. The results suggest that Wnt5a may be a target protein for EA to improve the ability of early pattern separation in AD mice.

## Conclusions

Our study has two main contributions. Firstly, we elucidate the significant role of Wnt5a in promoting improved pattern separation during neurogenesis. Secondly, our study further confirms and complements a previous study that EA may improve cognitive impairment by promoting neurogenesis [27]. Because we provide a molecular mechanism underlying the enhancement of memory discrimination ability in early AD model mice by EA treatment: EA regulates Wnt5a expression in the hippocampus of AD mice, which subsequently promotes neurogenesis in the adult hippocampal DG. The resulting generation of immature GCs mediate the recovery of pattern separation disorder in AD mice, ultimately leading to an improvement in their the memory discrimination ability. Furthermore, our study has certain limitations that warrant further investigation. For instance, the precise mechanism by which EA modulates the expression of Wnt5a remains unknown and will be a focus of our future research endeavors.

## Data Availability

The datasets used and/or analysed during the current study are available from the corresponding author on reasonable request.

## Abbreviations

5 × FAD: Ive familial AD mutations
AD: Alzheimer’s disease
GCs: Granule cells
DG: Dentate gyrus
EA: Electroacupuncture
OFT: Open field test
OLT: Object location task
TUNL: Touch Screen Unique Nonmatching to Location
AAV: Adeno-associated virus
Aβ: Amyloid-β
CaR: Calretinin
BrdU: 5-Bromo-2’-deoxyuridine
Prox1: Prospero homeobox1

## References

[1] Kronenberg G, Gertz K, Uhlemann R, Kuffner M, Kirste I, An J (2019). Reduced hippocampal neurogenesis in mice deficient in apoptosis repressor with caspase recruitment domain (ARC). Neuroscience.

[2] Wang H, Sun N, Wang X, Han J, Zhang Y, Huang Y (2022). A touchscreen-based paradigm to measure visual pattern separation and pattern completion in mice. Front Neurosci-Switz.

[3] Reagh ZM, Ho HD, Leal SL, Noche JA, Chun A, Murray EA (2016). Greater loss of object than spatial mnemonic discrimination in aged adults. Hippocampus.

[4] Stark SM, Yassa MA, Lacy JW, Stark CEL (2013). A task to assess behavioral pattern separation (BPS) in humans: data from healthy aging and mild cognitive impairment. Neuropsychologia.

[5] Riphagen JM, Schmiedek L, Gronenschild E, Yassa MA, Priovoulos N, Sack AT (2020). Associations between pattern separation and hippocampal subfield structure and function vary along th e lifespan: a 7T imaging study. Sci Rep.

[6] Maass A, Berron D, Harrison TM, Adams JN, La Joie R, Baker S (2019). Alzheimer’s pathology targets distinct memory networks in the ageing brain. Brain.

[7] Reagh ZM, Roberts JM, Ly M, DiProspero N, Murray E, Yassa MA (2014). Spatial discrimination deficits as a function of mnemonic interference in aged adults with and without memory impairment. Hippocampus.

[8] Snyder JS, Drew MR (2020). Functional neurogenesis over the years. Behav Brain Res.

[9] Gould E (2007). How widespread is adult neurogenesis in mammals?. Nat Rev Neurosci.

[10] Toda T, Parylak SL, Linker SB, Gage FH (2018). The role of adult hippocampal neurogenesis in brain health and disease. Mol Psychiatr.

[11] Yassa MA, Stark CE (2011). Pattern separation in the hippocampus. Trends Neurosci.

[12] Anacker C, Hen R (2017). Adult hippocampal neurogenesis and cognitive flexibility - linking memory and mood. Nat Rev Neurosci.

[13] Johnston ST, Shtrahman M, Parylak S, Gonçalves JT, Gage FH (2016). Paradox of pattern separation and adult neurogenesis: a dual role for new neurons balancing memory resolution and robustness. Neurobiol Learn Mem.

[14] Becker S (2017). Neurogenesis and pattern separation: time for a divorce. Wires Cogn Sci.

[15] França T, Bitencourt AM, Maximilla NR, Barros DM, Monserrat JM (2017). Hippocampal neurogenesis and pattern separation: a meta-analysis of behavioral data. Hippocampus.

[16] Sahay A, Scobie KN, Hill AS, O'Carroll CM, Kheirbek MA, Burghardt NS (2011). Increasing adult hippocampal neurogenesis is sufficient to improve pattern separation. Nature.

[17] Lazarov O, Marr RA (2010). Neurogenesis and Alzheimer's disease: at the crossroads. Exp Neurol.

[18] Babcock KR, Page JS, Fallon JR, Webb AE (2021). Adult hippocampal neurogenesis in aging and Alzheimer's disease. Stem Cell Rep.

[19] Sung PS, Lin PY, Liu CH, Su HC, Tsai KJ (2020). Neuroinflammation and neurogenesis in Alzheimer’s disease and potential therapeutic approaches. Int J Mol Sci.

[20] Chen CM, Orefice LL, Chiu SL, LeGates TA, Hattar S, Huganir RL (2017). Wnt5a is essential for hippocampal dendritic maintenance and spatial learning and memory in adult mice. P Natl Acad Sci USA.

[21] Varela-Nallar L, Alfaro IE, Serrano FG, Parodi J, Inestrosa NC (2010). Wingless-type family member 5A (Wnt-5a) stimulates synaptic differentiation and function of glutamatergic synapses. P Natl Acad Sci USA.

[22] Ortiz-Matamoros A, Arias C (2019). Differential changes in the number and morphology of the new neurons after chronic infusion of Wnt7a, Wnt5a, and Dkk-1 in the adult hippocampus in vivo. Anat Rec.

[23] Arredondo SB, Guerrero FG, Herrera-Soto A, Jensen-Flores J, Bustamante DB, Oñate-Ponce A (2020). Wnt5a promotes differentiation and development of adult-born neurons in the hippocampus by noncanonical Wnt signaling. Stem Cells.

[24] Zhang S, Jin T, Wang L, Liu W, Zhang Y, Zheng Y (2020). Electro-acupuncture promotes the differentiation of endogenous neural stem cells via exosomal microRNA 146b after ischemic stroke. Front Cell Neurosci.

[25] Pei W, Meng F, Deng Q, Zhang B, Gu Y, Jiao B (2021). Electroacupuncture promotes the survival and synaptic plasticity of hippocampal neurons and improvement of sleep deprivation-induced spatial memory impairment. CNS Neurosci Ther.

[26] Cai M, Lee JH, Yang EJ (2019). Electroacupuncture attenuates cognition impairment via anti-neuroinflammation in an Alzheimer's disease animal model. J Neuroinflamm.

[27] Li L, Li J, Dai Y, Yang M, Liang S, Wang Z (2021). Electro-acupuncture improve the early pattern separation in Alzheimer's disease mice via basal forebrain-hippocampus cholinergic neural circuit. Front Aging Neurosci.

[28] Zhao J, Kumar M, Sharma J, Yuan Z (2021). Arbutin effectively ameliorates the symptoms of Parkinson's disease: the role of adenosine receptors and cyclic adenosine monophosphate. Neural Regen Res.

[29] Hueston CM, O'Leary JD, Hoban AE, Kozareva DA, Pawley LC, O'Leary OF (2018). Chronic interleukin-1beta in the dorsal hippocampus impairs behavioural pattern separation. Brain Behav Immun.

[30] Kim CH, Romberg C, Hvoslef-Eide M, Oomen CA, Mar AC, Heath CJ (2015). Trial-unique, delayed nonmatching-to-location (TUNL) touchscreen testing for mice: sensitivity to dorsal hippocampal dysfunction. Psychopharmacology.

[31] Stergiopoulos A, Elkouris M, Politis PK (2014). Prospero-related homeobox 1 (Prox1) at the crossroads of diverse pathways during adult neural fate specification. Front Cell Neurosci.

[32] Karalay O, Doberauer K, Vadodaria KC, Knobloch M, Berti L, Miquelajauregui A (2011). Prospero-related homeobox 1 gene (Prox1) is regulated by canonical Wnt signaling and has a stage-specific role in adult hippocampal neurogenesis. P Natl Acad Sci USA.

[33] Kempermann G, Jessberger S, Steiner B, Kronenberg G (2004). Milestones of neuronal development in the adult hippocampus. Trends Neurosci.

[34] Barnard IL, Onofrychuk TJ, McElroy DL, Howland JG (2021). The touchscreen-based trial-unique, nonmatching-to-location (TUNL) task as a measure of working memory and pattern separation in rats and mice. Curr Protoc.

[35] McAllister KA, Saksida LM, Bussey TJ (2013). Dissociation between memory retention across a delay and pattern separation following medial prefrontal cortex lesions in the touchscreen TUNL task. Neurobiol Learn Mem.

[36] Talpos JC, McTighe SM, Dias R, Saksida LM, Bussey TJ (2010). Trial-unique, delayed nonmatching-to-location (TUNL): a novel, highly hippocampus-dependent automated touchscreen test of location memory and pattern separation. Neurobiol Learn Mem.

[37] Carrica L, Li L, Newville J, Kenton J, Gustus K, Brigman J (2019). Genetic inactivation of hypoxia inducible factor 1-alpha (HIF-1α) in adult hippocampal progenitors impairs neurogenesis and pattern discrimination learning. Neurobiol Learn Mem.

[38] Wesnes KA, Annas P, Basun H, Edgar C, Blennow K (2014). Performance on a pattern separation task by Alzheimer's patients shows possible links between disrupted dentate gyrus activity and apolipoprotein E in4 status and cerebrospinal fluid amyloid-beta42 levels. Alzheimers Res Ther.

[39] Jessberger S, Clark RE, Broadbent NJ, Clemenson GJ, Consiglio A, Lie DC (2009). Dentate gyrus-specific knockdown of adult neurogenesis impairs spatial and object recognition memory in adult rats. Learn Memory.

[40] Clelland CD, Choi M, Romberg C, Clemenson GD, Fragniere A, Tyers P (2009). A functional role for adult hippocampal neurogenesis in spatial pattern separation. Science.

[41] Kempermann G, Song H, Gage FH (2015). Neurogenesis in the adult hippocampus. CSH Perspect Biol.

[42] Nakashiba T, Cushman JD, Pelkey KA, Renaudineau S, Buhl DL, McHugh TJ (2012). Young dentate granule cells mediate pattern separation, whereas old granule cells facilitate pattern completion. Cell.

[43] Lavado A, Lagutin OV, Chow LM, Baker SJ, Oliver G (2010). Prox1 is required for granule cell maturation and intermediate progenitor maintenance during brain neurogenesis. PLoS Biol.

[44] Bengoa-Vergniory N, Kypta RM (2015). Canonical and noncanonical Wnt signaling in neural stem/progenitor cells. Cell Mol Life Sci.

[45] As B, Daniela VB, MM D, Lorena VN (2020). Role of Wnt signaling in adult hippocampal neurogenesis in health and disease. Front Cell Dev Biol.

[46] Freese JL, Pino D, Pleasure SJ (2010). Wnt signaling in development and disease. Neurobiol Dis.

[47] Wang Y, Li YP, Paulson C, Shao JZ, Zhang X, Wu M (2014). Wnt and the Wnt signaling pathway in bone development and disease. Front Biosci-Landmrk.

[48] Zou Y, Salinas P (2014). Introduction: Wnt signaling mechanisms in development and disease. DEV NEUROBIOL.

[49] Rim EY, Clevers H, Nusse R (2022). The Wnt pathway: from signaling mechanisms to synthetic modulators. Annu Rev Biochem.

[50] Riise J, Plath N, Pakkenberg B, Parachikova A (2015). Aberrant Wnt signaling pathway in medial temporal lobe structures of Alzheimer's disease. J Neural Transm.

[51] De Ferrari GV, Papassotiropoulos A, Biechele T, Wavrant DF, Avila ME, Major MB (2007). Common genetic variation within the low-density lipoprotein receptor-related protein 6 and late-onset Alzheimer's disease. P Natl Acad Sci USA.

[52] Tay L, Leung B, Yeo A, Chan M, Lim WS (2019). Elevations in serum Dickkopf-1 and disease progression in community-dwelling older adults with mild cognitive impairment and mild-to-moderate Alzheimer's disease. Front Aging Neurosci.

[53] Rosi MC, Luccarini I, Grossi C, Fiorentini A, Spillantini MG, Prisco A (2010). Increased Dickkopf-1 expression in transgenic mouse models of neurodegenerative disease. J Neurochem.

[54] Caraci F, Busceti C, Biagioni F, Aronica E, Mastroiacovo F, Cappuccio I (2008). The Wnt antagonist, Dickkopf-1, as a target for the treatment of neurodegenerative disorders. Neurochem Res.

[55] Bischoff DS, Zhu JH, Makhijani NS, Yamaguchi DT (2015). Induction of CXC chemokines in human mesenchymal stem cells by stimulation with secreted frizzled-related proteins through non-canonical Wnt signaling. World J Stem Cells.

[56] Schafer ST, Han J, Pena M, von Bohlen UHO, Peters J, Gage FH (2015). The Wnt adaptor protein ATP6AP2 regulates multiple stages of adult hippocampal neurogenesis. J Neurosci.

[57] Wang LY, Pei J, Zhan YJ, Cai YW (2020). Overview of meta-analyses of five non-pharmacological interventions for Alzheimer's disease. Front Aging Neurosci.

[58] Huang Q, Luo D, Chen L, Liang FX, Chen R (2019). Effectiveness of acupuncture for Alzheimer's disease: an updated systematic review and meta-analysis. Curr Med Sci.

[59] Kim JH, Cho MR, Shin JC, Park GC, Lee JS (2021). Factors contributing to cognitive improvement effects of acupuncture in patients with mild cognitive impairment: a pilot randomized controlled trial. Trials.

[60] Lin R, Li L, Zhang Y, Huang S, Chen S, Shi J (2018). Electroacupuncture ameliorate learning and memory by improving N-acetylaspartate and glutamate metabolism in APP/PS1 mice. BIOL RES.

[61] Jeon H, Ryu S, Kim D, Koo S, Ha KT, Kim S (2017). Acupuncture stimulation at GB34 restores MPTP-induced neurogenesis impairment in the subventricular zone of mice. Evid-Based Compl Alt.

[62] Zheng X, Lin W, Jiang Y, Lu K, Wei W, Huo Q (2021). Electroacupuncture ameliorates beta-amyloid pathology and cognitive impairment in Alzheimer disease via a novel mechanism involving activation of TFEB (transcription factor EB). Autophagy.

